# Crustal architecture of a metallogenic belt and ophiolite belt: implications for mineral genesis and emplacement from 3-D electrical resistivity models (Bayankhongor area, Mongolia)

**DOI:** 10.1186/s40623-021-01400-9

**Published:** 2021-04-01

**Authors:** Matthew J. Comeau, Michael Becken, Alexey V. Kuvshinov, Sodnomsambuu Demberel

**Affiliations:** 1 Institut für Geophysik, Universität Münster, Corrensstrasse 24, 48149 Münster, Germany; 2 Institute of Geophysics, Swiss Federal Institute of Technology (ETH), Sonneggstrasse 5, 8092 Zürich, Switzerland; 3 Institute of Astronomy and Geophysics, Mongolian Academy of Sciences, P.O.B-152, Ulaanbaatar, 13343 Mongolia

**Keywords:** Metallogenic belt, Ophiolite belt, Fault zone, Mineralization, Mineral emplacement, Electrical resistivity

## Abstract

Crustal architecture strongly influences the development and emplacement of mineral zones. In this study, we image the crustal structure beneath a metallogenic belt and its surroundings in the Bayankhongor area of central Mongolia. In this region, an ophiolite belt marks the location of an ancient suture zone, which is presently associated with a reactivated fault system. Nearby, metamorphic and volcanic belts host important mineralization zones and constitute a significant metallogenic belt that includes sources of copper and gold. However, the crustal structure of these features, and their relationships, are poorly studied. We analyze magnetotelluric data acquired across this region and generate three-dimensional electrical resistivity models of the crustal structure, which is found to be locally highly heterogeneous. Because the upper crust (< 25 km) is found to be generally highly resistive (> 1000 Ωm), low-resistivity (< 50 Ωm) features are conspicuous. Anomalous low-resistivity zones are congruent with the suture zone, and ophiolite belt, which is revealed to be a major crustal-scale feature. Furthermore, broadening low-resistivity zones located down-dip from the suture zone suggest that the narrow deformation zone observed at the surface transforms to a wide area in the deeper crust. Other low-resistivity anomalies are spatially associated with the surface expressions of known mineralization zones; thus, their links to deeper crustal structures are imaged. Considering the available evidence, we determine that, in both cases, the low resistivity can be explained by hydrothermal alteration along fossil fluid pathways. This illustrates the pivotal role that crustal fluids play in diverse geological processes, and highlights their inherent link in a unified system, which has implications for models of mineral genesis and emplacement. The results demonstrate that the crustal architecture—including the major crustal boundary—acts as a first‐order control on the location of the metallogenic belt.

**Supplementary Information:**

The online version contains supplementary material available at 10.1186/s40623-021-01400-9.

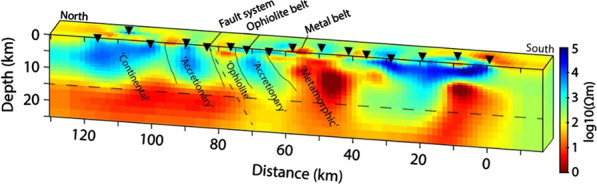

## Introduction

A long ophiolite belt in the Bayankhongor region of central Mongolia marks the location of a prominent, crustal-scale, ancient suture zone that is today associated with a re-activated fault system (e.g., Walker et al. 2007; Badarch et al. 2002; Buchan et al. 2001). Adjacent to this region is a significant metallogenic belt that includes important sources of gold and copper (see Gerel et al. 2021) hosted in metamorphic and volcanic belts (Osozawa et al. 2008; Buchan et al. 2001), which are common settings for ore deposits (e.g., Groves et al. 2018; Yardley and Cleverley 2013). Although many active mineral deposits are known here, their crustal structure, as well as their geological history, is poorly studied.

Links between the locations of mineralized zones and their underlying crustal structure—at both small and large scales—have been established for many well-known mineral districts (e.g., Heinson et al. 2006) and thus knowledge of the structural framework can lead to a better understanding of the development of the mineral system. In fact, through analyzing the spatial (and temporal) distribution of mineral ore systems, it has been recognized that locations near significant crustal boundaries, specifically those with a history of tectonic/geodynamic processes and crustal reworking, may be crucial in the formation of mineralized zones (e.g., Groves et al. 2018; Huston et al. 2016), and that their internal geometry is inherited from earlier tectonic features (Groves et al. 2018). Indeed, the mineral systems concept, whereby mineralized zones are seen as small expressions of a range of Earth processes, is acknowledged as the key to targeting new deposits and to interpreting ore genesis, through understanding the organizational framework of the system (e.g., Davies et al. 2020).

In this study, we analyze geophysical data from magnetotelluric (MT) measurements and generate three-dimensional (3-D) images of the electrical resistivity structure beneath the Bayankhongor region. We determine and describe the geometry and extent of the metallogenic belt and of the fault system and suture zone, which are associated with the ophiolite belt. Based on our results, we attempt to shed light on potential implications for the genesis and emplacement of the mineral systems within the metallogenic belt.

## Regional background

**Fig. 1:**
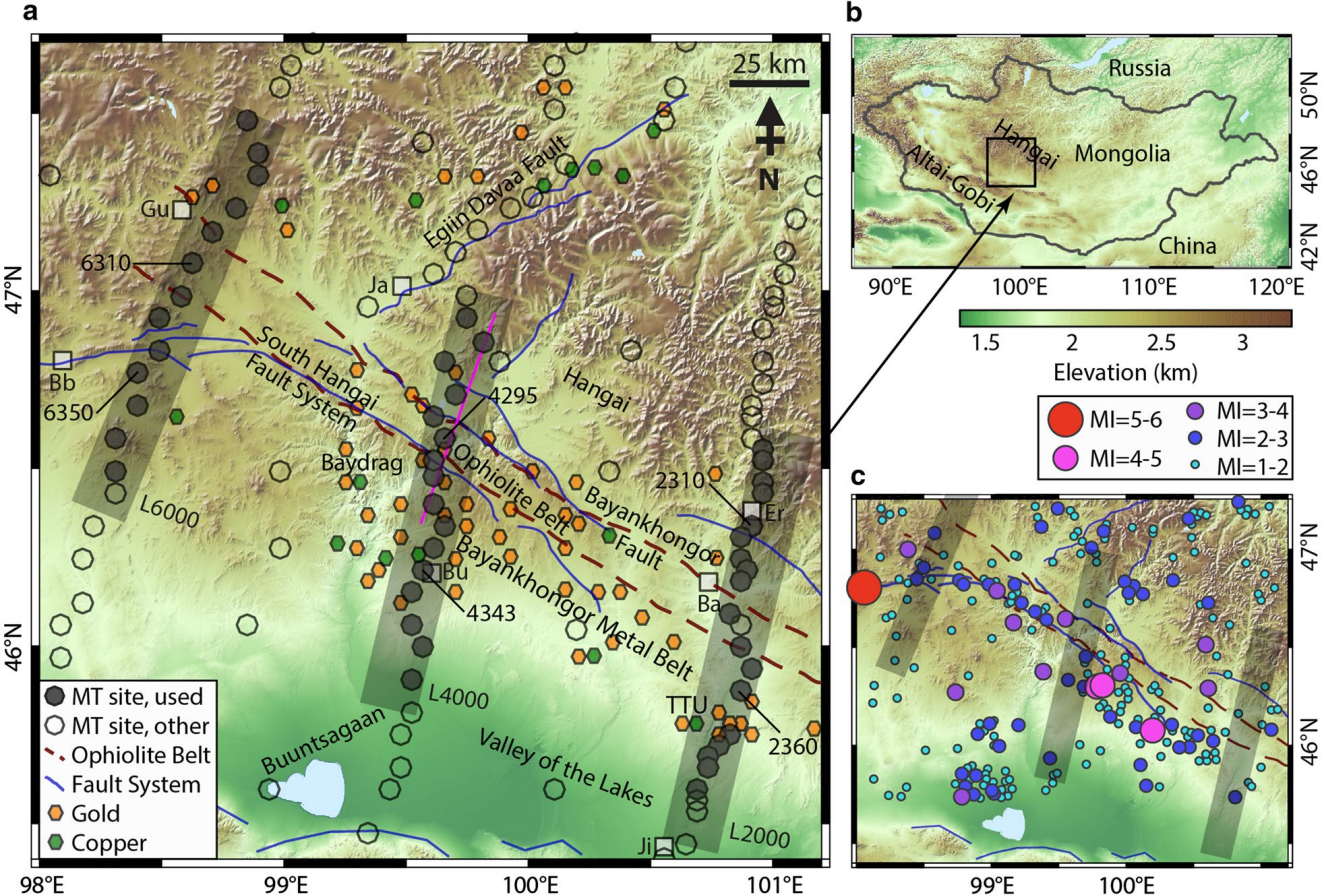
Topographic map of the study area. **a** The locations of the MT measurement sites are indicated with black circles; filled circles are used in this study. Each profile is marked with a thick grey line (L2000; L4000; L6000). Specific site names are identified (see Fig. 4). Faults, including the South Hangai fault system, are marked (solid blue lines; Walker et al. 2007; Styron et al. 2018). The approximate location of the Bayankhongor Ophiolite Belt is shown (red dashed lines; Osozawa et al. 2008; Badarch et al. 2002). The location of the geological transect of Osozawa et al. (2008), congruent with L4000, is marked in pink (see Fig. 2). This region contains many mineralized zones, which constitute the Bayankhongor Metal Belt, that contain significant occurrences of copper and gold, identified with green and yellow hexagons, respectively (Mineral Resources Authority of Mongolia 2017; Dejidmaa and Badarch, 1999). Villages are labeled for reference (white boxes; Gu: Gurvanbulag; Bb: Bayanbulag; Ja: Jargalant; Bu: Bumbugur; Er: Erdenestsogt; Ba: Bayankhongor; Ji: Jinst). **b** The survey area in a regional context. **c** Distribution of recent seismicity (2012 to 2014; data from Dashdondog et al. 2020)

**Fig. 2:**
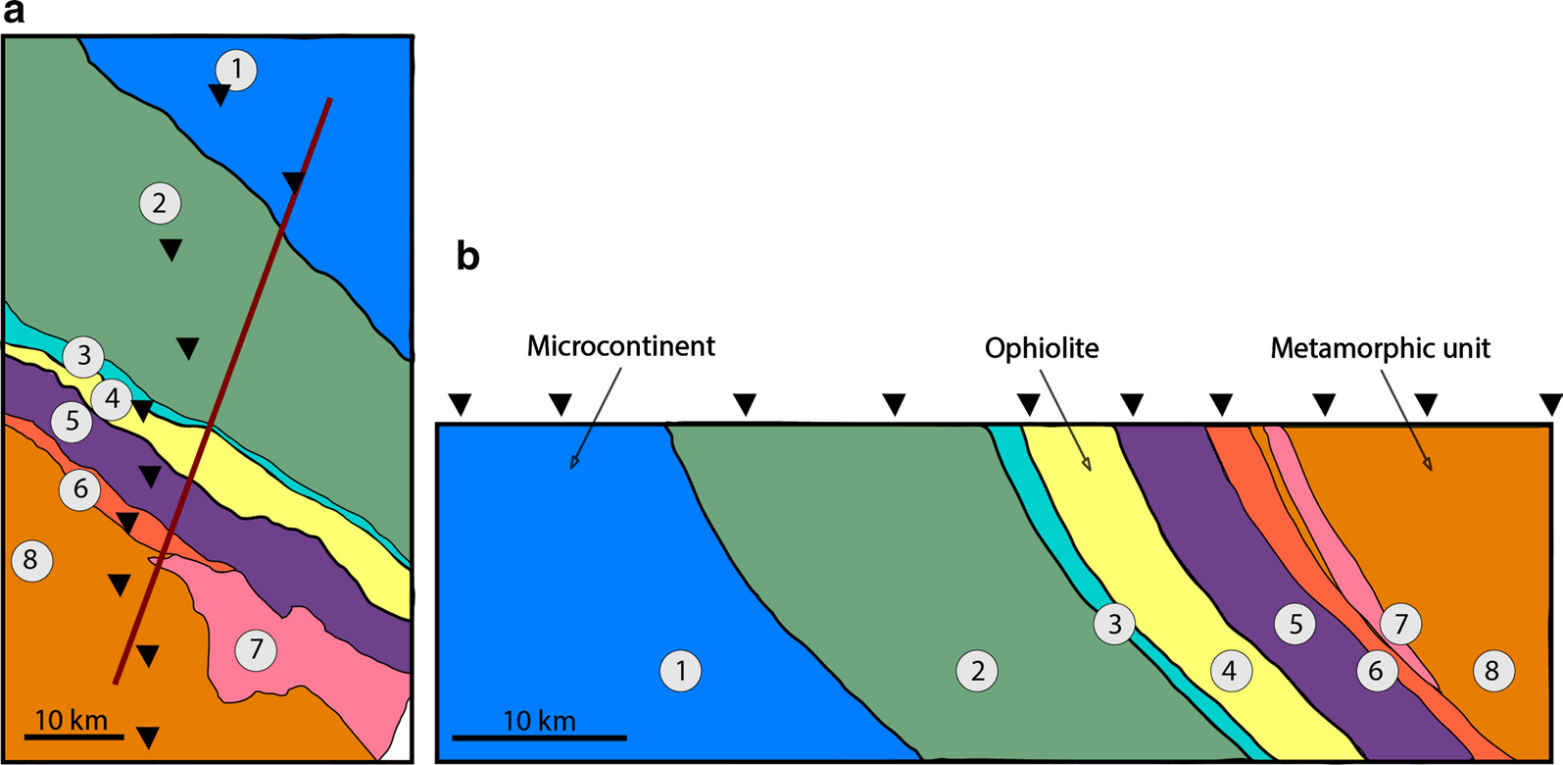
Simplified geological map (**a**) and geological cross-section (**b**) (modified from Osozawa et al. 2008). The geological cross-section is coincident with profile L4000; its location is marked with a line on the map (see also Fig. 1). The locations of MT measurement sites are indicated with black triangles (along L4000). Several south-dipping lithotectonic units are identified. These include: (1) Hangai microcontinent; (2) Dzag metamorphic continental margin; (3) Haluut Bulg accretionary complex; (4) Bayankhongor Ophiolite Belt; (5) Delb Khairkhan accretionary complex; (6) Ulaan Sair volcanic unit; (7) Granite unit; (8) Burd Gol metamorphic unit

In central Mongolia, the location of a large suture zone, a consequence of the collision of distinct tectonic blocks, is marked by the Bayankhongor Ophiolite Belt (Badarch et al. 2002; Buchan et al. 2001; Cunningham 2001) (Figs. 1, 2). Its significance lies in the fact that ophiolite belts, which can be defined as oceanic lithosphere thrust over a continental margin, can give evidence for the subduction-related closure of paleo-oceans (e.g., Dilek and Furnes 2011). This region lies within the Central Asian Orogenic Belt, an accretionary orogeny that covers a large area of Central and Eastern Asia (e.g., Yin 2010).

The origin and evolution of this part of the Central Asian Orogenic Belt is complex and remains controversial (e.g., Kovach et al. 2013; Kröner et al. 2010), but its tectonic history is known to include subduction events and ocean closures (e.g., Jiang et al. 2017; Xiao et al. 2015; Van der Voo et al. 2015; Windley et al. 2007). The mechanism of Palaeozoic continental growth in the Central Asian Orogenic Belt is believed to have been by subduction and accretion (e.g., Kröner et al. 2010; Buchan et al. 2002). However, it is somewhat unique because there is evidence for a punctuated style of accretion, in which multiple episodes of discrete collisional events produced sutures between accreted blocks (and possibly corresponding ophiolite belts), rather than gradual and steady-state subduction and accretion over a prolonged period of time (Buchan et al. 2002; see also Goldfarb et al. 2014).

Today, the Bayankhongor Ophiolite Belt is located at the southern margin of the Hangai Mountains and the northern margin of the Valley of the Lakes, a depression north of the Gobi-Altai mountains. It is possibly the longest continuously exposed ophiolite belt in Asia, with a length of 300 km and a width of up to 20 km (Buchan et al. 2002). It is believed to have been formed prior to and during the Cambrian (Buchan et al. 2002, 2001). In addition, there is evidence that implies subduction may have been occurring for long (> 100 million years) before the obducted ophiolitic rocks were formed (Buchan et al. 2001).

Buchan et al. (2002) suggest that, during an episode of subduction–accretion, the ophiolite belt was accreted to an accretionary complex, subsequently obducted onto the passive continental margin of the Dzag zone, part of the Hangai block, and later deformed by a protracted collisional event (lasting into the Silurian). The result was the suturing of (Proterozoic) microcontinental blocks: the Baydrag block (to the south) and the Dzag complex and Hangai block (to the north). In addition, southward subduction of oceanic lithosphere, and the following plate rollback, resulted in the development of a metamorphic and volcanic belt south of the suture zone (Zhang et al. 2015). Its development and evolution may have been further complicated by reworking and consecutive events which may have been overprinted on the aforementioned structures, and the later (Cenozoic) collision of the Indian and Eurasian continents that resulted in significant far-field deformation (e.g., Calais et al. 2003).

Segments of the South Hangai fault system, which is a ~ 350-km-long shear system (identified as left-lateral strike–slip), pass through this area (Walker et al. 2007; Calais et al. 2003). They are believed to be part of a reactivated fault system (Walker et al. 2007) from pre-existing weaknesses and show considerable small-scale seismicity, both historically and recently (Welkey et al. 2018; Adiya et al. 2003). This is in contrast to the large, continuous strike–slip faults that lie to the north and south of the Hangai region that are known for their large but infrequent intracontinental ruptures (Rizza et al. 2015, 2011; Calais et al. 2003). Remote sensing, ground measurements, and seismicity are used to map the position of the fault system (Welkey et al. 2018; Walker et al. 2007) and it is closely correlated with the location of the ancient ophiolite belt. Therefore, the system is believed to represent an important crustal boundary (and rheological boundary) between distinct tectonic blocks (Badarch et al. 2002; Buchan et al. 2001). This is confirmed by evidence that indicates significant lateral seismic velocity variations across the system (Welkey et al. 2018).

To the south, metamorphic and (mafic) volcanic belts are found (Osozawa et al. 2008) that contain significant mineral deposits and make up the Bayankhongor Metal Belt (see Fig. 1; Jargalan et al. 2007; Buchan et al. 2001; Dejidmaa and Badarch, 1999; Watanabe et al. 1999). Distributed throughout this region are notable occurrences of gold and copper mineralization, in addition to iron ore (see Gerel et al. 2021). Deposits are commonly composed of gold-bearing quartz veins and porphyry-type copper (Watanabe et al. 1999). Many questions remain as to the structure of these mineral zones, which could shed light on their origins (see Groves et al. 2018), including their near-surface framework, their vertical extent, their possible connection to deeper sources, and their relationship to other crustal features.

The genesis of these mineral zones is closely connected to the unique and complex tectonic history of this area, which created good metallogenic conditions (see Goldfarb et al. 2014). The age of the mineralization in this area is younger than that of the ophiolite belt—they date mostly to the Permian or late Palaeozoic (Jargalan et al. 2007; Jargalan and Fujimaki 2000; Watanabe et al. 1999). They may correspond temporally to melting or magmatic events after subduction when a high regional compressional stress was present, possibly a critical component, due to the collisional deformation of the Baydrag microcontinent (Watanabe et al. 1999). Their formation may be analogous to the major ore-producing regions of north-eastern Mongolia that are closely linked to a major suture zone (see Goldfarb et al. 2014). An explanation for the difference in ages between mineralization episodes and preceding subduction events, proposed by Goldfarb and Santosh (2014), hypothesizes that fluids from the devolatilization of a subducting slab flow up-dip and temporarily reside in the mantle wedge where they significantly enrich and hydrate the lithosphere, which later releases ore-forming fluids due to successive heating or the cessation of subduction (Goldfarb and Groves 2015).

Seismic studies indicate that the crust beneath central Mongolia is thick (approximately 45 km to 50 km) and that the lithosphere is anomalously thin (approximately 60 km to 80 km), compared to its surroundings (which may be more than 150 km thick) (Petit et al. 2008; Tiberi et al. 2008; Stachnik et al. 2014). However, the detailed 3-D seismic structure of central Mongolia, including the shallow crust, is currently unknown. Recently, the lithospheric-scale regional 3-D resistivity model of Käufl et al. (2020), generated from MT data, revealed a complex asthenospheric upwelling beneath central Mongolia with links to crustal features, including an anomalously conductive lower crust. Comeau et al. (2020b) demonstrated that this implies a weak, low-viscosity lower crust. The geophysical results are consistent with geochemical evidence from central Mongolia that indicates the lower crust is hotter than normal (Ionov et al. 1998), and that melts are generated in the shallow mantle (Barry et al. 2003). They are also compatible with a previous lithospheric removal event that brought hot, buoyant material to shallow depths, as explored by Becker et al. (2019) and others, which is supported by geochemical data (Sheldrick et al. 2020b). In this study, we employ the MT method to characterize the electrical resistivity distribution in the Bayankhongor region and to investigate the geometry and extent of features, as well as their relationships.

## The magnetotelluric method

The MT method is a geophysical technique used to probe the subsurface electrical structure of the Earth. It uses passive electromagnetic signals generated in the atmosphere and ionosphere over a broad range of frequencies (e.g., Unsworth and Rondenay 2012). MT data consist of electric and magnetic fields measured at the Earth's surface. These fields are related by a frequency-dependent, complex-valued impedance tensor (*Z*) that is sensitive to the subsurface electrical resistivity structure. Apparent resistivity (*ρ*) and impedance phase (*φ*) are then determined over a range of frequencies (e.g., Chave and Jones 2012). Additionally, the ratio of horizontal and vertical magnetic fields, the tipper, can be included in MT data analysis and interpretation. The high-frequency (short period) data are sensitive to shallow structures and the low-frequency (long period) data probe deep structures; hence the technique can be used over various spatial scales.

Studies have shown that the MT technique is ideally suited to detect and image the structure of faults and suture zones (e.g., Karas et al. 2017; Türkoğlu et al. 2014, 2008; Becken et al. 2011, 2008; Unsworth and Bedrosian 2004; Comeau et al. 2020a), which are regions of fractured and weakened crust due to past and present deformation (e.g., Dewey 1977). This is because the technique is especially sensitive to the quantity and composition of fluids, which reduce electrical resistivity (e.g., Unsworth and Rondenay 2012; Chave and Jones 2012). Although subduction zones have been extensively studied with the MT technique for similar reasons (e.g., Reyes-Wagner et al. 2017; Soyer and Unsworth 2006; Brasse and Soyer 2001), there has been much less work done in obducted environments with ophiolite belts (see Thiel et al. 2009). In addition, numerous studies have shown that the MT technique is capable of characterizing the pathways of past fluids and the traces of alteration (i.e., relic metasomatism), including those involved in the formation of mineral ore deposits (e.g., Heinson et al. 2018, 2006; Pritchard et al. 2018; Hübert et al. 2015; Comeau et al. 2015). This is due to the fact that the presence of a reasonable amount of alteration products and ore-associated material (such as sulfide, e.g., < 1%) can cause a strong electrical signature (e.g., Pearce et al. 2006).

### Data analysis

The MT data analyzed here are a subset of an extensive dataset (total of 334 measurement sites) collected across central Mongolia since 2016 (Käufl et al. 2020; Comeau et al. 2020a, 2020b, 2018a). Here we focus on three profile segments (Line 2000, Line 4000, and Line 6000) located south of the Hangai Mountains in central Mongolia (Fig. 1). The profiles are approximately 120 km long and are each separated by approximately 100 km. They consist, respectively, of 18, 16, and 13 measurement sites (total of 47), and thus have an average site spacing of approximately 6.5 km, 7.5 km, and 9.0 km (note the minimum site spacing is > 4 km). A combination of broadband MT instruments (SPAM Mk IV data-loggers and Metronix MFS-06e magnetic induction coils; provided by the Geophysical Instrument Pool Potsdam) and telluric recorders (typically ~ 60 m electric dipole length) were deployed (see details in Käufl et al. 2020). Data collection at each site was typically carried out over several days and was recorded at a sampling frequency of 512 Hz. The MT data are generally high quality and have a very low noise level. This is primarily due to the remote location and lack of cultural noise. Nevertheless, careful data editing was performed to remove any spurious data points and outliers.

A dimensionality analysis of the data determines the validity of the assumption of a two-dimensional (2-D) Earth model, typical for large regional surveys. If this assumption is not locally true, then a 3-D analysis may be required to properly model the data. Phase tensors, which can be graphically represented as ellipses, are commonly used for dimensionality analysis (Caldwell et al. 2004; Booker 2014). They will appear circular for a one-dimensional (1-D) subsurface and elliptical for 2-D or 3-D subsurface. The ellipse axes are parallel to the directions of greatest and least inductive response, and thus are analogous to directions along and across geo-electric strike in 2-D modeling (i.e., when the skew angle is 0°; Bibby et al. 2005). The phase tensor skew angle can also be determined (Caldwell et al. 2004), and the ellipses can be colored with their skew value (Booker 2014). Large-magnitude skew values indicate significant 3-D effects, whereas low values (e.g., less than 3°) imply a 2-D assumption is reasonable.

**Fig. 3:**
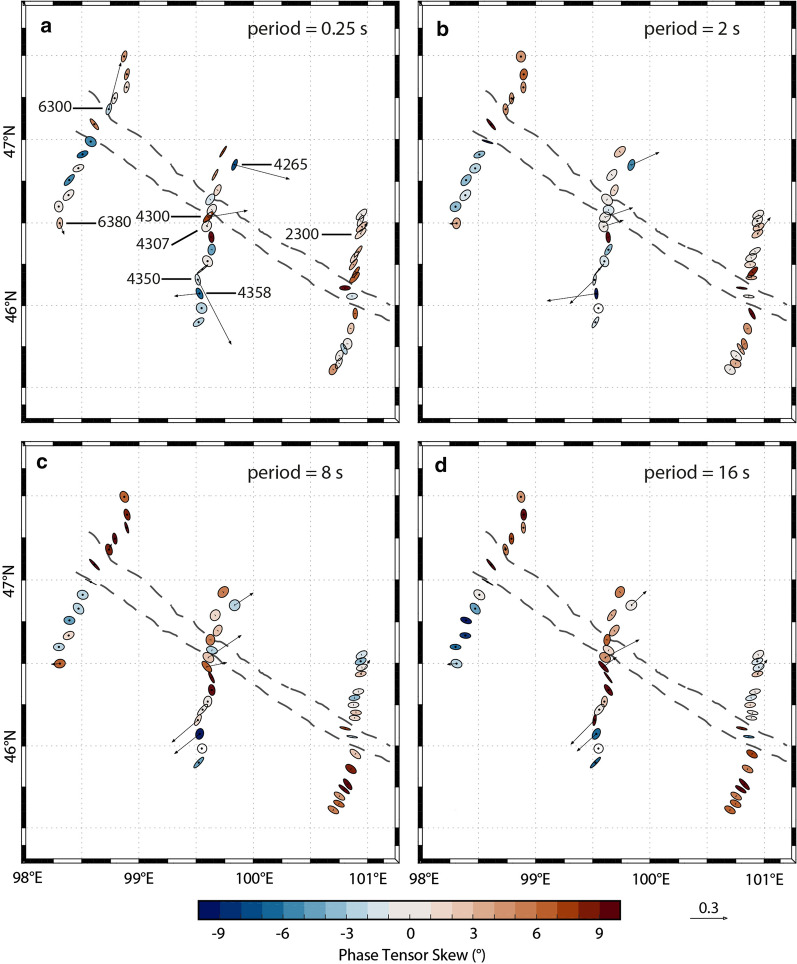
Phase tensor ellipses computed from the data. They are shown in map view for selected periods of approximately **a** 0.25 s, **b** 2 s, **c** 8 s, and **d** 16 s. They have been normalized by their maximum axis values and are colored with their skew values. Elongated ellipses and large absolute skew values indicate possible 3-D effects. The periods correspond to depths of approximately 2 km, 7 km, 14 km, and 21 km, respectively, from simple skin depth estimations. Induction vectors (arrows) are computed from the tipper data (site names are marked); they point away from conductors (i.e., Wiese convention). A scale arrow of magnitude 0.3 is shown. The approximate location of the Bayankhongor Ophiolite Belt is marked (dashed lines, as in Fig. 1). This feature appears to influence the phase tensors

For this dataset, phase tensor analysis shows many large skew values (Fig. 3). For periods of ~ 0.25 s to 16 s, which corresponds to a range of depths within the crust of approximately 2 km to 21 km (based on skin depth estimation), the median skew values are in the range of 3° to 6°. Most phase tensor ellipses appear to be aligned with the ophiolite belt (see Fig. 3; e.g., Line 2000 at both 8 s and 16 s). This indicates that it is a major conductive structure. However, on Line 4000, the phase tensors that lie south of the ophiolite belt are somewhat scattered, and may be influenced by near-surface conductive features (e.g., mineralized zones). An interesting variation in phase tensor skew values is detected across the ophiolite belt, with values changing from high to low (e.g., on Line 6000), possibly reflecting a transition in crustal properties. Some ellipses within the ophiolite belt are highly elongated and are parallel to the belt. These observations are indications of local 3-D resistivity structures, as may be expected in a complicated faulted and mineralized setting, and suggest that a full 3-D inversion algorithm is required to model the data.

### Data modeling

The MT data along three profiles were inverted with the MODEM inversion algorithm (Kelbert et al. 2014; Egbert and Kelbert 2012) and 3-D electrical resistivity models were generated. Data included the full impedance tensor (off-diagonal and diagonal components, which were similar in magnitude and both smooth) and the tipper data. Note the tipper data were not available at every site (and were not inverted in the regional model of Käufl et al. 2020). Because of the large separation between profiles (approximately 100 km), it was found that separate models for each profile allowed for much smaller models with a finer discretization of the modeling grid, and consequently the possibility to better resolve more detailed structure, as well as faster and smoother convergence, compared to one model for all the profiles combined. Similar model parameters were chosen for each profile.

Because the target of this study was the upper crust, data to a period of 0.01 s was included in the inversion and the longest period was limited to 1024 s, unlike the regional model of Käufl et al. (2020) that focused on longer period data from 0.1 s to ~ 20,000 s. The starting model consisted of a homogenous 100 Ωm halfspace, which was based on the mean value for the entire region determined by a previous 1-D analysis (see Käufl et al. 2020). The modeling grids were rotated so that the model coordinate system was parallel to the profiles and so that the modeling domain was reduced in size; the profiles had angles of 11°, 11°, and 22°, for Line 2000, Line 4000, and Line 6000. No topography was included in the models (the terrain in this region is not particularly rough). Model depths are given as depth below surface, which in this region is approximately 2000 m above sea-level (consult Fig. 1). The impedance components Zxy and Zxx were assigned an error floor of 5% of |Zxy|, and similarly the components Zyx and Zyy were assigned an error floor of 5% of |Zyx| (cf. approach of Käufl et al. 2020). This corresponds to a minimum error of 2.86° on impedance phase. The tipper components were assigned an absolute error floor of 0.05.

The models used a modeling grid of rectangular elements (cells). Within the area of interest, the (column) width of the horizontal grid elements was fixed to 1400 m. This is approximately 15% to 25% of the average measurement site spacing, within the range of commonly used values reported by Robertson et al. (2020), and also a few times smaller than the minimum site spacing. This size should be chosen with careful consideration to the numerical accuracy of the forward solution, and thus depends on the (smallest) period of data used and the (smallest) electrical resistivity (anticipated)—that is, on the smallest skin depth (see Weaver 1994). The (relatively small) cell size gave the model the ability to resolve fine and detailed structures, at least in comparison to the regional model of Käufl et al. (2020); although the regional model employed local refinement near each site, it achieved a minimum cell width larger (approximately twice as large) than the fixed column width used here. The thickness (row height) of the vertical grid elements smoothly increased with depth, by a factor of 1.1, from an initial thickness of 100 m to a total depth of 500 km. This corresponds to a cell thickness of less than 1 km for all depths less than 10 km, which maintains the ability to image fine structures in the upper crust. Cell elements have a thickness of less than 1.9 km for depths less than 20 km, whereas for depths larger than 33 km the cells have thicknesses of more than 3.1 km, which means that the models lose detail. In addition to the elements within the modeling domain of interest, seven padding elements were added in each direction for numerical stabilization of the inversion algorithm (see Weaver 1994). The horizontal padding cells expanded smoothly by a factor of 2, which increased the model boundaries by ~ 400 km each. The total number of cells for each model differed slightly but were 94 to 103 along the profile (*x*-direction), 30 to 32 across the profile (*y*-direction), and 66 vertically (*z*-direction) (see Table 1 for details). The forward and inverse modeling grids were the same.

**Table 1:** Details of each 3-D inversion model

Line	# of sites	Starting misfit	Final misfit	# of iterations	# cells in model
Line 2000	18	16.79	2.008	110	94 × 30 × 66
Line 4000	16	17.23	1.815	99	100 × 31 × 66
Line 6000	13	10.18	1.294	92	103 × 32 × 66

For each profile line, information is provided including the number of sites, the starting model RMS misfit, the final model RMS misfit, the total number of iterations, and the number of cells used in the model (Nx × Ny × Nz)

Many inversion setups were investigated in order to test the robustness of the model features. This included testing the model covariance and the resistivity of the starting (and prior) model (see Additional file 1 for a selection of modeling trials). Choosing the model covariance (between 0 and 1) is a trade-off between overly smooth and unrealistically coarse models and affects both the final model structure and resistivity values (see Robertson et al. 2020, for detailed comparisons; see Comeau 2015, and references therein). A covariance value of 0.5 was preferred for the horizontal directions and for the vertical direction (fixed) after testing various values (see Additional file 1). The choice of the resistivity structure of the starting model was determined to have a moderate influence on the final model, although the main structures remained consistent. A starting model consisting of a homogenous halfspace of 100 Ωm was preferred over other values or 1-D layered models (see Additional file 1). Overall, these model tests suggested that the main resistivity features of the preferred models were representative of most cases, and that the main features were required, particularly the uppermost crustal features (< 15 km depth) that were well resolved and where model sensitivity was found to be high.

**Fig. 4:**
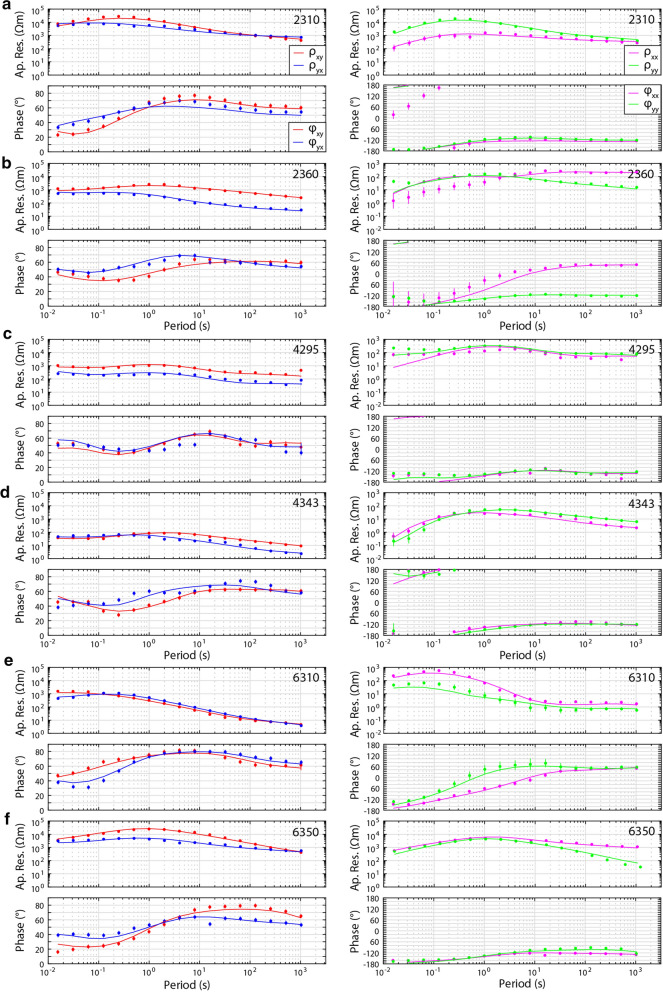
Magnetotelluric data (dots) and model response (solid lines). They are shown as apparent resistivity (Ap. Res.; *ρ*) and phase (*φ*) from all impedance components (off-diagonal, xy and yx, left column; diagonal, xx and yy, right column) at six selected (representative) sites. The locations of the measurement sites are indicated in Fig. 1 (site names: 2310; 2360; 4295; 4343; 6310; 6350). Assigned error floors are shown as error bars (see text for details). In general, the models fit the data

**Fig. 5:**
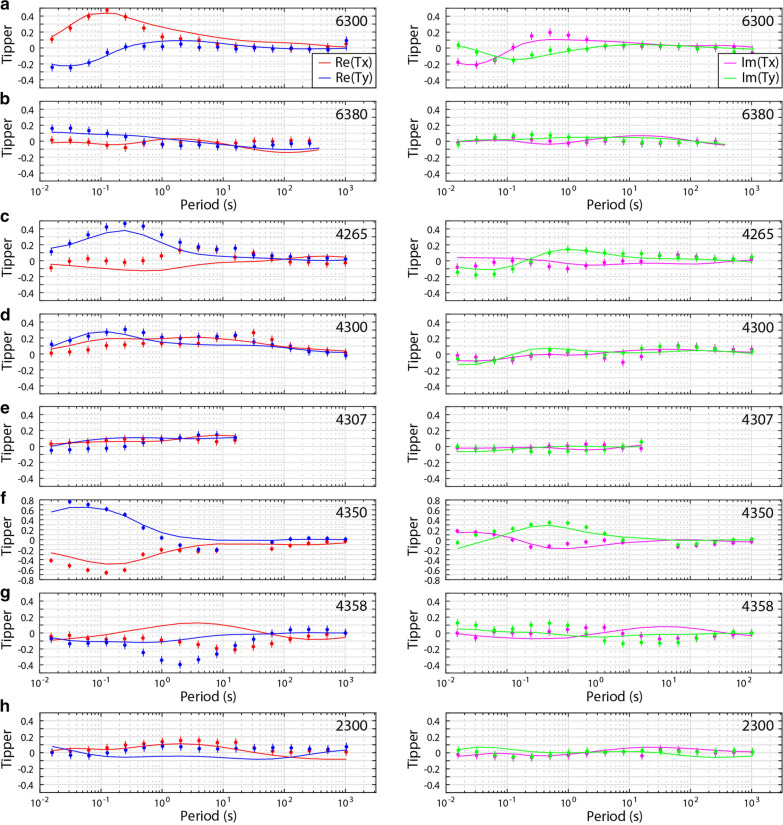
Tipper data (dots) and model response (solid lines). They are shown as Tx and Ty components with real parts (left columns) and imaginary parts (right columns) at eight sites. The locations of the measurement sites correspond to the locations of the induction vectors shown in Fig. 2 (site names: 6300; 6380; 4265; 4300; 4307; 4350; 4358; 2300). The tipper components were assigned an absolute error floor of 0.05

**Fig. 6:**
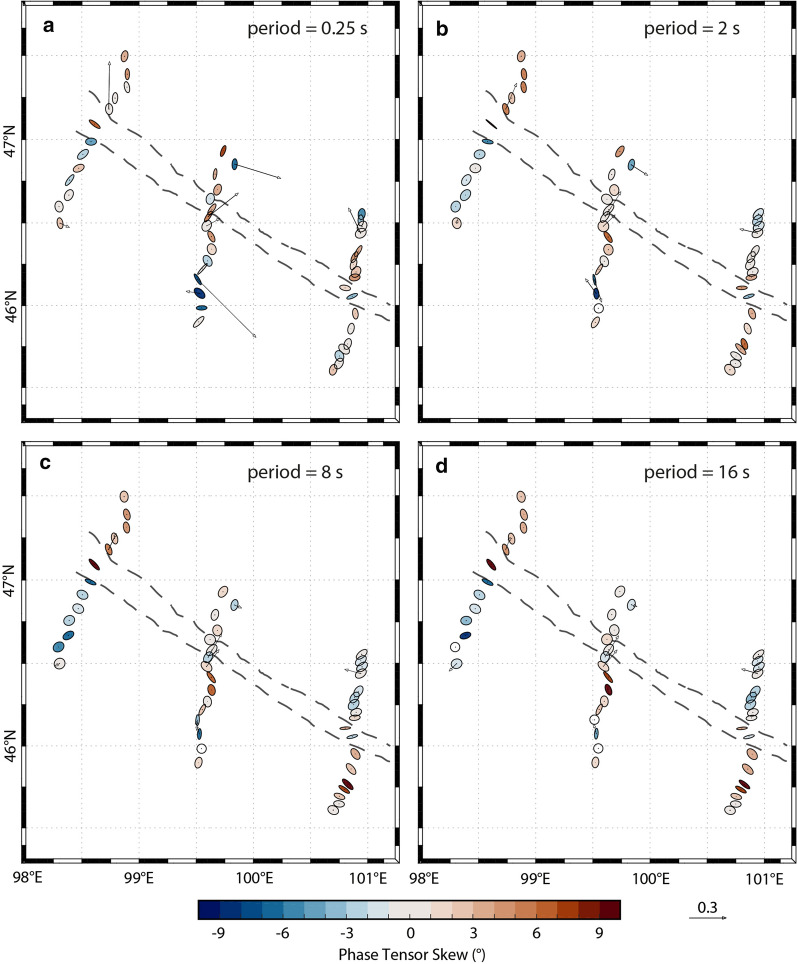
Phase tensor ellipses computed from the models. They are shown in map view for selected periods of approximately **a** 0.25 s, **b** 2 s, **c** 8 s, and **d** 16 s. They have been normalized by their maximum axis values and are colored with their skew values. Induction vectors (arrows) are computed from the tipper data; they point away from conductors (i.e., Wiese convention). A scale arrow of magnitude 0.3 is shown. The approximate location of the Bayankhongor Ophiolite Belt is marked (dashed lines, as in Fig. 1). The modeled phase tensors show ellipse shapes and orientations as well as skew magnitudes and signs that are very similar to the phase tensors from the data (see Fig. 3). This indicates that the models fit the data

For each model, Line 2000, Line 4000, and Line 6000, the inversion algorithm smoothly converged after 110, 99, and 92 iterations, respectively, and the root-mean-square (RMS) misfit was reduced to 2.0, 1.8, and 1.3 (from a starting model with a misfit of approximately 17, 17, and 10), respectively (see Table 1 for details). This suggests that the final models fit the data (i.e., approximately within their errors bars), but examining only the RMS misfit criterion is not sufficient to determine overall model fit (e.g., Grayver et al. 2013). Careful comparisons of the measured data and the modeled data show that the models fit the data for the off-diagonal and diagonal impedance components and for the tipper. Furthermore, the data fit is fairly equally spread across all the measurement sites, suggesting that no part of the data are unfairly influencing the model. Similarly, the data fit appears to be reasonably equally distributed between data components. See Fig. 4 for the data fit of six selected (representative) sites (2310; 2360; 4295; 4343; 6310; 6350) and Fig. 5 for the fit of tipper data at eight sites (6300, 6380, 4265; 4300; 4307; 4350; 4358; 2300); also the Additional file 1 includes data from four more sites (4335; 4300; 4290; 4285). In addition, the phase tensors of the model responses (Fig. 6) show very similar shapes and orientations to the phase tensors of the data; their skew values also match well, in both sign and magnitude. This indicates that the models fit the data adequately.

## Modeling results

**Fig. 7:**
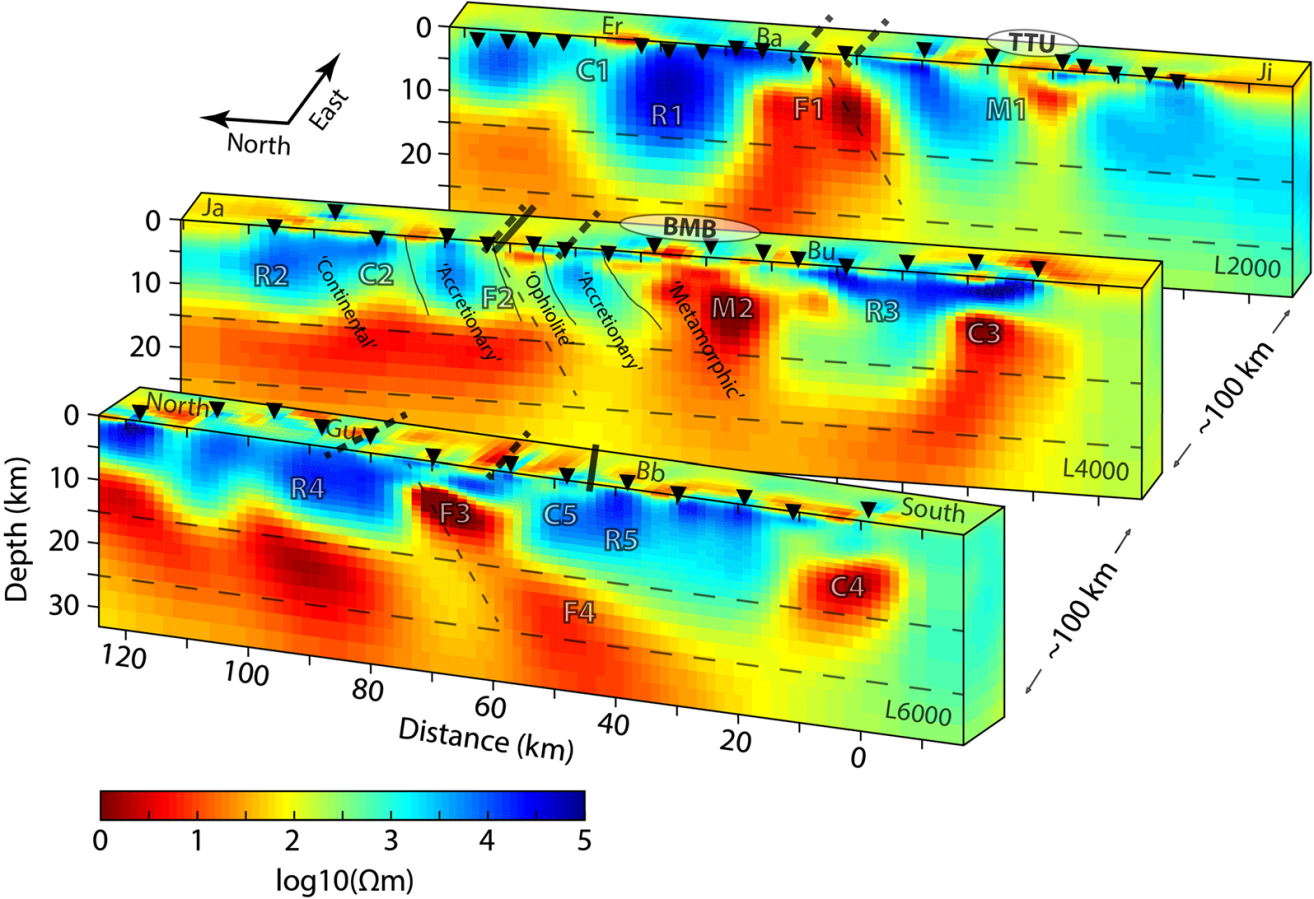
The preferred 3-D electrical resistivity models. The models, along three profiles (L2000, L4000, and L6000), were obtained with the MODEM inversion algorithm (Kelbert et al. 2014). The locations of MT measurement sites are indicated with black triangles. Distances are along the profiles, from south to north, along longitudes of approximately 98.5 °E, 99.7 °E, and 100.7 °E (see Fig. 1). Black lines correspond to the South Hangai fault system (solid) and to the Bayankhongor Ophiolite Belt (dashed; see Fig. 1), which are related to a major crustal boundary and ancient suture zone. Anomalous features in the upper crust appear coincident with surface fault/suture positions (e.g., F1, F2, and F3). Fault/suture extension in the subsurface (dashed grey lines) is speculated, and is intended only to illustrate down-dip features. The locations of several prominent mineralized zones are marked (BMB: Bayankhongor Metal Belt; TTU: Tsagaan Tsahir Uul and Saran Uul). They are coincident with low-resistivity anomalies in the shallow upper crust (M1 and M2). Highly resistive features in the north are attributed to a continental block north of the ophiolite belt and suture zone (Hangai; R1, R2, and R4). Conductive features C3 and C4 are of unknown origin. Geological units, and their contacts, are labeled on profile L4000 from the congruent cross-section of Osozawa et al. (2008). Villages are labeled as in Fig. 1. Note that the crust is believed to be 45 km to 50 km thick. Horizontal dashed lines denote the approximate depths of the upper crust, midcrust, and lower crust

**Fig. 8:**
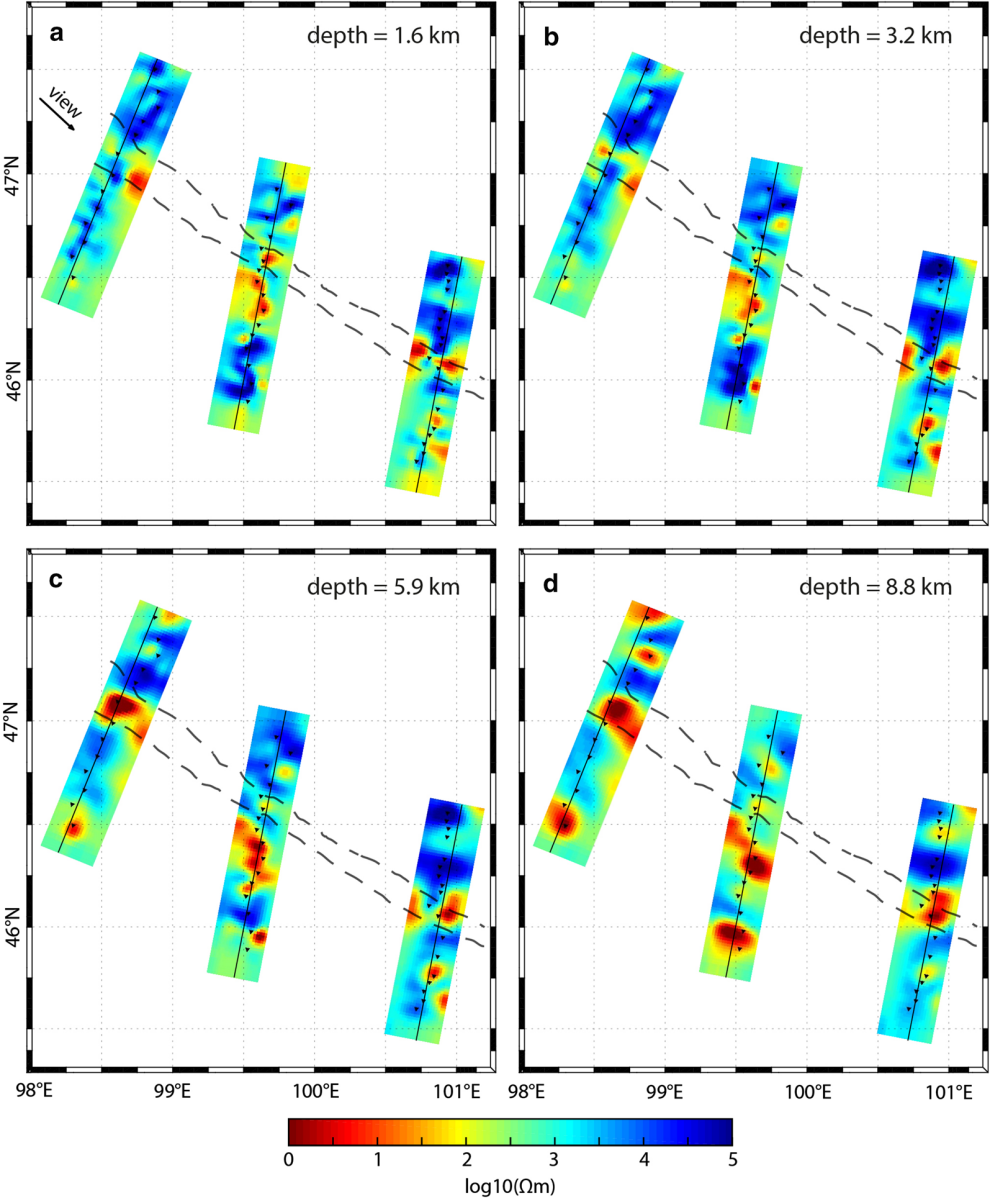
Horizontal slices of the 3-D electrical resistivity models. They are shown in map view for depths of approximately **a** 1.6 km, **b** 3.2 km, **c** 5.9 km, and **d** 8.8 km. The models were obtained with the MODEM inversion algorithm. The locations of MT measurement sites are indicated with black triangles. The approximate location of the Bayankhongor Ophiolite Belt is marked (dashed lines, as in Fig. 1). An arrow marks the direction of the oblique view from Fig. 6

The 3-D resistivity models (Figs. 7,  8) image a crust that is heterogeneous and contains several prominent features, which are discussed below. For convenience, we label features as follows: resistive features with an R; general conductive features with a C; specific conductive features associated with the ophiolite belt and the suture/fault zone with an F; specific conductive features associated with known mineralization (and a metamorphic belt) with an M. Significantly, the features found in the local 3-D inversions discussed here, which focus on the upper crust, broadly match the shallow features described in the lithospheric-scale regional model of Käufl et al. (2020), although direct comparison is not straightforward due to differences in model discretization, particularly in the uppermost crust. Because the models use different modeling grids and inversion algorithms, as well as different data subsets and different periods, this adds credibility to the results. Variations in resistivity can have several explanations, including different rock types, the presence of fluids, hydrothermal alteration, or the presence of partial melts (e.g., Unsworth and Rondenay 2012). Therefore, an interpretation of the features benefits from additional information.

In general, the uppermost portion of the crust (< 15 km) appears highly resistive (> 1000 Ωm), with some areas exceeding 10,000 Ωm. This is explained by the ancient (Precambrian) microcontinental Hangai and Baydrag blocks (Badarch et al. 2002; Cunningham 2001). In contrast, the midcrust and lower crust appear to have a much lower resistivity (approximately 100 Ωm to 300 Ωm). The near-surface layer is highly variable (e.g., 10 Ωm to 1000 Ωm) and is likely controlled by surface sediments. The most prominent features are strongly conductive (i.e., they have a low resistivity; < 50 Ωm) structures imaged throughout the crust, some of which stretch nearly vertically from the surface to lower-crustal depths, whereas others appear localized near the top of the upper crust with only moderate links to mid-crustal depths. The features are well resolved and constrained because of the included high-frequency data in combination with the dense and regular measurement site spacing and the finely discretized modeling grid.

The location of the Bayankhongor Ophiolite Belt, which is along a suture system (Osozawa et al. 2008; Badarch et al. 2002; Buchan et al. 2001), and the surface trace of the South Hangai fault system (believed to be reactivated), which is often spectacularly exposed at the surface (Walker et al. 2007; Calais et al. 2003), are coincident with several of the conductive anomalies. The locations of other conductive anomalies match with the locations of mineralized zones within the Bayankhongor Metal Belt (Mineral Resources Authority of Mongolia 2017; Jargalan et al. 2007; Dejidmaa et al. 2002; Buchan et al. 2001; Dejidmaa and Badarch 1999; Watanabe et al. 1999).

### Line 2000

Along Line 2000, a moderate anomaly (~ 300 Ωm; C1, see Fig. 7) is detected near the village of Erdenestsogt, which stretches downwards through the resistive upper crust. This is believed to be related to the young (< 1 million years ago) intraplate volcanism found at this location (Ancuta et al. 2018), which was one of several volcanic environments imaged by Comeau et al. (2018b). South of this anomaly, a highly resistive feature (> 1000 Ωm; R1) is imaged. It is thought to represent the ancient microcontinental block (Hangai block).

The resistive upper crust is interrupted by a large conductive anomaly (< 50 Ωm; F1) south of the village of Bayankhongor. It appears to be nearly vertical, extends to depths of approximately 30 km, or more, and widens with depth. The location of the large conductive anomaly F1 is closely correlated with the inferred location of the ophiolite belt. It is also aligned with the general trend of the South Hangai fault system, which is not mapped continuously at this location. Potential field methods (e.g., magnetics or gravity) could elucidate structural boundaries for these features, providing more precise locations than current geological mapping (Guy et al. 2014; see also Comeau et al. 2020a). Note that depths of approximately 20 km to 25 km correspond to a rheological transition from brittle to ductile crust in this region (Welkey et al. 2018; Li et al. 2017; Calais et al. 2003; Déverchère et al. 2001), and that the crust is assumed to be 45 km to 50 km thick. Conductive anomalies embedded in the moderately resistive lower crust can be attributed to metamorphic fluids trapped below the brittle–ductile transition, as demonstrated by Comeau et al. (2020b) based on a conceptual model of hydrodynamic stagnation of crustal fluids from Connolly and Podladchikov (2004).

Further south, an isolated conductive anomaly (< 10 Ωm; M1) appears at a depth of < 7 km and comes to the surface at its northern end. The shape and size is consistent with that reported by Comeau et al. (2020a), based on 2-D modeling of a transect across the Gobi-Altai mountains. This feature lies beneath a mineralized zone at the eastern extension of the Bayankhongor Metal Belt, near the well-studied Tsagaan Tsahir Uul region that includes the Saran Uul gold-bearing copper porphyry deposit along the Tuin Gol (Jargalan et al. 2007; Jargalan and Fujimaki 2000; Watanabe et al. 1999). Geologically, this area is described as a Proterozoic metamorphic unit with adjacent granites (Jargalan et al. 2007). Below this anomaly is a moderately resistive feature extending to the midcrust. Southwards, the crust becomes again resistive.

### Line 4000

At the northern end of Line 4000 (south of Egiin Davaa), a highly resistive feature (> 1000 Ωm; R2) is imaged. This is interpreted to be the ancient microcontinental block (Hangai block). The coincident geological cross-section of Osozawa et al. (2008) shows several south-dipping lithotectonic units (based on structure and composition) in this region, including accretionary complexes around the Bayankhongor Ophiolite Belt, a granitic unit, and volcanic, metamorphosed, and mineralized units to the south (see Fig. 2). A set of thrust faults separate the lithotectonic units (Buchan et al. 2002). These contrasting geological materials correspond to observed resistive and conductive features.

A moderate conductor (~ 100 Ωm; C2) is located near the inferred location of a fault at the south end of the microcontinental block (see map in Fig. 1). A clear conductive feature (< 50 Ωm; F2) is congruent with the ophiolite belt and possibly also with the South Hangai fault system. It appears to be dipping slightly southwards (approximately 60° to 80° to the horizontal), consistent with geological dip estimates (e.g., Buchan et al. 2001), although its shape is perhaps not robust (see Additional file 1 for possible model variations). This geometry is distinct, as expected, from the flower-structure features that joined at depths of 10 km to 20 km identified by Comeau et al. (2020a) for fault systems in southern Mongolia, confirming geological conjecture (e.g., Vassallo et al. 2007). Adjacent to the fault system and within the ophiolite belt, a strong (< 10 Ωm; near F2), shallow (< 3 km) anomaly is imaged that outcrops at the surface. It is located in the Duck Canyon zone (south of Darvsin Nuur and along the Uldzit Gol), which hosts a gold deposit. Further south, a strong conductive feature (< 10 Ωm; M2) appears at a depth of < 15 km. It is located within a highly metamorphosed unit (Osozawa et al. 2008; Buchan et al. 2001), and beneath the Bayankhongor Metal Belt, near the village of Bumbugur. Interestingly, this region also has heightened seismicity (see Fig. 1c), typically occurring at depths of 5 km to 15 km (see Dashdondog et al. 2020), that may be attributed to small, reactivated faults and pre-existing weaknesses within the metamorphic belt, which facilitated previous metasomatism. It should be noted that ruptures along faults lead to enhanced permeability (e.g., Sibson 2020).

Further south a highly resistive feature is imaged (> 1000 Ωm; R3). Beneath this a strong conductor (< 10 Ωm; C3) is observed at depths > 7 km. It does not reach the surface along the profile, but may come near the surface to the east. This feature is unexplained, however, it appears somewhat similar to M2. In the regional model of Käufl et al. (2020), the feature is imaged clearly and connects to another crustal structure to the south. Furthermore, near this location (close to the brackish Buuntsagaan lake), there has been a cluster of heightened seismicity detected in the upper crust (Adiya et al. 2003), with an unknown origin, that may be related.

### Line 6000

To the west, along Line 6000, the north end of the profile again shows a highly resistive feature (> 1000 Ωm; R4). To the south of the village of Gurvanbulag, a strong conductive anomaly (< 10 Ωm; F3) is imaged at a depth of < 10 km, and is linked to a larger dipping anomaly (F4) that extends through the crust and widens with depth. This feature is within the bounds of the ophiolite belt, and its dip direction and angle is consistent with geological estimates. The ophiolite belt and suture zone is expected to end near this point (Badarch et al. 2002; Buchan et al. 2001). In fact, it may mark the hinge point of an ocean closure event (e.g., comparable to the Mongol-Okhotsk Ocean closure that created a prominent suture zone across much of Mongolia, east of this region; see Van der Voo et al. 2015; see also Sheldrick et al. 2020a). The resistivity model suggests that the suture zone may extend slightly further west than the profile, which is along a longitude of approximately 98.5 °E, although perhaps it is not fully exposed at the surface, and hence has not been confirmed by other data, but is an open question.

Further south, a moderate conductor (< 100 Ωm; C5) is detected near the location of the trace of the South Hangai fault, which changes trend and bends from a north-west direction to a westerly direction and passes near the village of Bayanbulag. However, this feature does not appear to be strong and does not appear to be connected to the surface trace of the fault. This region has had recent seismic activity, including earthquake events of magnitude > 4 since 2012 (Dashdondog et al. 2020; Welkey et al. 2018; Adiya et al. 2003; see Fig. 1c). This feature may be connected, at a depth of 5 km to 10 km, to the larger south-dipping conductive feature (i.e., F3).

At the south end of the profile, the upper crust becomes again highly resistive (> 1000 Ωm; R5). This is interrupted by a new, isolated conductive feature (< 30 Ωm; C4). It is observed at depths of approximately 7 km to 18 km and does not reach the surface. Its origin is unknown at this time.

## Discussion and implications

The spatial distribution and emplacement of mineralized zones is mainly influenced by crustal architecture, structure and formation, which is partly controlled by tectonic/geodynamic processes, because it has an impact on the flow of fluids throughout the crust (e.g., structurally enhanced permeability; Huston et al. 2016). Thus the crustal features and structural framework inferred in this study, from electrical resistivity data, can shed light on the development of the mineral systems within the Bayankhongor Metal Belt. Note that other geophysical parameters such as magnetism and gravity—or their gradients—can also elucidate crustal boundaries, as can strain gradient identification, and that joint analysis of distinct results can lead to more information (e.g., Motta et al. 2019; Groves et al. 2018; Guy et al. 2014).

The electrical resistivity models in this study show that some conductive anomalies align with crustal boundaries, such as fault and suture zones. These may be explained by the fact that such regions consist of fractured and weakened crust and often contain fluids that act to increase the electrical conductivity, either directly or with their byproducts (e.g., Unsworth and Rondenay 2012; Chave and Jones 2012).

Throughout this region (and the Hangai Mountains), meteoric fluids are detected at geothermal hot springs (Oyuntsetseg et al. 2015), and are likely circulating in the near-surface. In contrast, mid-crustal conductors associated with faults and tectonic boundaries may be explained by hydrothermal alteration along fossil fluid pathways, which the MT method is sensitive enough to detect (e.g., Comeau et al. 2016). These fluids may have been sourced through metamorphic dehydration reactions in the crust (see Yardley and Cleverley 2013), with buoyant fluid propagating upwards, controlled by the permeability distribution (Cox 2002), as hypothesized in other mineral systems (Drummond et al. 2004). This is in agreement with geochemical evidence from central Mongolia that indicates a hot lower crust (Ionov et al. 1998). Note that the alignment of earthquake hypocenters along the edges of the observed conductive zones could act as a clear indicator of their internal weakness (e.g., Ogawa and Honkura 2004). Furthermore, in southern Mongolia, structural features and boundaries throughout the crust are shown to be related to past deformation episodes associated with previous accretion and subduction events (Guy et al. 2015) that may have widely hydrated and metasomatized the lithosphere (see Sheldrick et al. 2018, 2020b).

Interestingly, the strong conductive anomalies closely correlated with the location of the ophiolite belt appear to extend through the entire crust (e.g., see feature F4). This is consistent with the idea that the suture zone may be crustal or lithospheric in scale because it represents a major boundary (Calais et al. 2003; Badarch et al. 2002). Such structures may be maintained, despite reworking and consecutive tectonic events over time, due to reactivation (see Motta et al. 2019).

The deep structure of faults, especially in the ductile crust, is an open research topic (e.g., Türkoğlu et al. 2014; Becken et al. 2008). The broad conductive zones that lie down-dip from the fault and suture traces (in the midcrust and lower crust) in this region suggest that the narrow deformation zone observed at the surface and in the shallow brittle crust transforms to dispersed shear deformation across a wide area in the deeper ductile crust. This observation may shed light on much-contested theories about the evolution of faulting within the lithosphere (e.g., Wilson et al. 2004).

Some conductive anomalies match closely with the locations of mineralized zones within the Bayankhongor Metal Belt. This region contains many gold deposits as well as copper deposits, which often form together (e.g., Müller and Groves 2016; Goldfarb et al. 2014), hosted within belts of metamorphosed materials (Osozawa et al. 2008; Jargalan et al. 2007; Buchan et al. 2001). In general, mineralized zones commonly have large bulk conductivity signatures, which likely arise from emplacement-related metamorphic processes, including metasomatism (i.e., alteration), and from the associated sulfide mineralogy (e.g., precipitation), even within small-scale mineral deposits. Gold-bearing mineralization, in particular, may be formed by hydrothermal alteration from fluid–rock interactions, which are greatly enhanced by crustal deformation processes (e.g., Goldfarb et al. 2014; Yardley and Cleverley 2013; Sibson 2007; Cox et al. 1999).

Throughout the Bayankhongor area there exists evidence of extensive hydrothermal alteration (Jargalan et al. 2007; Buchan et al. 2001; Watanabe et al. 1999). Fluid inclusion analysis of gold-bearing quartz veins in the metal belt indicate an apparent fluid salinity of ~ 4 wt% NaCl and mineral deposition at temperatures ranging from 160 °C to 260 °C (Jargalan and Fujimaki 2000; Jargalan and Murao 1998). These are typical values for similar ore-bearing environments—although some caution is advised as this type of analysis may be equivocal (e.g., due to possible post-entrapment processes; Goldfarb and Groves 2015; Zhu et al. 2011). Notably, numerous lamprophyre dikes (ultrapotassic mafic igneous intrusives) are found within the gold-bearing and copper-bearing zones (Jargalan et al. 2007; Jargalan and Fujimaki 2000). Such dikes are known to be both spatially and temporally correlated with gold mineralization and deeply-connected, ore-forming fluid pathways (Groves et al. 2018)—and are likely formed as a consequence of elevated fluid flow through the crust during subduction-related metamorphism (Müller and Groves 2016; see also Goldfarb and Santosh 2014; Groves et al. 2018), as is believed to have occurred in this region. Overall, the evidence indicates that the conductive signatures observed are due to hydrothermal fluid alteration related to ore-emplacement processes.

We hypothesize that the genesis and emplacement of the minerals within the metallogenic belt is directly connected to the unique and complex tectonic history of the Bayankhongor region. Considering the available evidence—including the history of subduction and obduction as well as metamorphism, the location of the ophiolite belt, and the difference in ages between the ophiolite belt (suture zone) and the mineralization zones—we favor a model of mineral emplacement that hypothesizes that fluids generated by devolatilization within a subducting slab flow up-dip along the plate boundary to the mantle wedge and hydrate the lithosphere (Goldfarb and Groves 2015; Goldfarb and Santosh 2014; see also Davies et al. 2020), and that later enriched ore-forming fluids move to the upper crust, preferentially along pre-existing weaknesses and pathways (including the plate boundary, suture zone, and shallow thrusts faults) (Goldfarb and Groves 2015; Yang and Santosh 2020; Drummond et al. 2004). The results illustrate that crustal architecture, specifically significant crustal boundaries including crustal (and lithospheric)-scale faults and suture zones that are inherited from earlier tectonic events, acts as a first‐order control on the locations of mineral deposits and metallogenic belts, due to its influence on (ore-related) fluid pathways (see Motta et al. 2019; Groves et al. 2018).

## Conclusions

The 3-D electrical resistivity models presented here, generated from magnetotelluric data, provide new insights into the electrical resistivity structure of the Bayankhongor area and its crustal architecture, from which we can derive implications for mineral emplacement and origin. Low-resistivity anomalies (< 50 Ωm) are revealed throughout the crust. The locations of some are found to be closely correlated with the Bayankhongor Ophiolite Belt, which marks a paleo-ocean closure and is related to an ancient suture zone between tectonic blocks. Notably, it is revealed to be a deep-reaching structure and a major crustal boundary. It is also spatially associated with the South Hangai fault system, a reactivated fault system with recent seismicity. Down-dip from the fault and suture traces the conductive zones broaden. This indicates that the narrow deformation zone observed in the shallow brittle crust may transform to a wide zone in the deeper ductile crust, as shear deformation is dispersed. The locations of other low-resistivity anomalies are spatially coincident with known mineralized zones in the Bayankhongor Metal Belt, which contains significant copper and gold deposits hosted in metamorphic and volcanic belts, to the south of the suture zone.

Considering the evidence revealed by the electrical resistivity models in combination with geological and petrological data, we conclude that, in both cases, the low resistivity observed can be explained by hydrothermal alteration along fossil fluid pathways. We suggest that there is a fundamental and inherent link between the imaged features, with paleo-subduction processes and crustal fluids playing a pivotal role in both cases. Ultimately, we speculate that a model of mineral emplacement in which devolatized fluids within the down-going slab flow up-dip and at a later time enriched, ore-forming fluids move through the crust along pre-existing weaknesses and fault systems can explain the available data. Overall, the results demonstrate that crustal architecture, specifically major crustal boundaries, acts as a first‐order control on the locations of mineral deposits and metallogenic belts.

## Supplementary Information

**Additional file 1: Figure S1.** Varying the starting model for model L4000. Panels (a), (b), (c), and (d) show the inversion model results when using an initial model that is a halfspace of 10, 100, 300, and 1,000 Ωm, respectively. Panels (e) and (f) use layered initial models, based on averaging the 2-D model of Comeau et al. (2018a) and from averaging 1-D models below each site, respectively. The model in (b) is the preferred model; it fits the data best. Note these models use a vertical increase factor of 1.2, and a horizontal covariance parameter of 0.5. Boxes highlight main features. Details for each model are (starting model RMS misfit / number of iterations / final RMS misfit): (a) 12.25 / 96 / 2.186, (b) 17.22 / 120 / 1.588, (c) 27.75 / 96 / 1.936, (d) 51.98 / 86 / 2.008, (e) 33.16 / 94 / 2.031, (f) 19.54 / 88 / 1.824. **Figure S2. **Varying the horizontal covariance parameter for model L4000. Panels (a), (b), (c), (d), and (e) show the inversion model results for a horizontal covariance parameter of 0.3, 0.4, 0.5, 0.6, and 0.7, respectively. Large values smooth the model. Note the effect of the covariance is over the number of cells rather than the physical cell size, see Robertson et al. (2020). The model in (c) is the preferred model; it fits the data best. Note these models use a vertical increase factor of 1.2 (38 vertical cells), as well as a starting model of a 100 Ωm halfspace. The vertical covariance parameter was fixed, as in (a). Details for each model are as follows (starting model RMS misfit / number of iterations / final model RMS misfit): (a) 17.22 / 75 / 2.247, (b) 17.22 / 105 / 1.968, (c) 17.22 / 120 / 1.588, (d) 17.22 / 114 / 1.903, (e) 17.22 / 93 / 2.498. **Figure S3. **Varying the data included for model L4000. The inversion model results shown in panel (a) include full impedance (off-diagonal and diagonal components) and tipper data, (b) full impedance data (off-diagonal and diagonal components), and (c) the off-diagonal components (Zxy, Zyx) of the impedance only. Note these models use a vertical increase factor of 1.2 (38 vertical cells), a starting model of a 100 Ωm halfspace, as well as a horizontal covariance parameter of 0.5. It is clear that the inclusion of the diagonal components influences the model and modifies the shape and strength of some structures. However, the locations of the main model features (boxes), and their interpretations, do not change. Details for each model are as follows (starting model RMS misfit / number of iterations / final model RMS misfit): (a) 17.22 / 120 / 1.588, (b) 18.45 / 95 / 1.757, (c) 21.92 / 78 / 1.227. **Figure S4. **Comparing the fit of six sites for model L4000 (apparent resistivity, ρapp, and phase, φ). Panels (a), (b), (c), (d), (e), and (f) correspond to sites 4343, 4335, 4300, 4295, 4290, and 4285 from the model (Figure S3c) that includes only off-diagonal components (column i) and the model (Figure S3b) that includes both off-diagonal and diagonal components (column ii). The corresponding diagonal components are shown in panels (g), (h), (i), (j), (k), and (l). The sites chosen are those above features of interest that appear to change between the two models (i.e., the fault/suture zone and the mineral belt). In both cases the fit of the model to the data is good. It is clear that the inclusion of the diagonal components influences the fit of the off-diagonal components. **Figure S5. **Error-normalized residuals of eight sites for model L4000. The residuals were computed as the absolute difference of the modelled data from the measured data for both apparent resistivity (top part) and phase (bottom part). Panels (a), (b), (c), (d), (e), (f), (g), and (h) correspond to sites 4377, 4343, 4335, 4300, 4295, 4290, 4285, and 4257 from the model (Figure S3c) that includes only off-diagonal components (column i) and the model (Figure S3b) that includes both off-diagonal and diagonal components (column ii). Note that sites 4377 and 4257 are at the ends of the model, not above the features of interest. The gray region indicates values <1.5, i.e. the absolute difference is less than 1.5x the error. The RMS misfit is given for each part. The residuals illustrate clearly that both models fit the data. Comparing the two models, it is obvious that there are differences in the residuals at all sites (not only those above the features of interest). Although the misfit is generally (slightly) higher at all sites for the model that includes both off-diagonal and diagonal components, the fit is still good.

## Data Availability

The MT data are archived by the German Research Centre for Geosciences (GFZ) Potsdam. For details about access visit the Data Services portal through the GIPP Experiment Database.

## Abbreviations

MT: Magnetotelluric
1-D: One-dimensional
2-D: Two-dimensional
3-D: Three-dimensional
RMS: Root mean square
