# Supplementary material for: Crustal architecture of a metallogenic belt and ophiolite belt: implications for mineral genesis and emplacement from 3-D electrical resistivity models (Bayankhongor area, Mongolia)
Source: Earth Planets Space. 2021 Apr 1;73(1):82. doi: 10.1186/s40623-021-01400-9 (PMC8550322; doi:10.1186/s40623-021-01400-9)
Supplement: Supplementary file 1 — Additional file 1: Figure S1. Varying the starting model for model L4000. Panels (a), (b), (c), and (d) show the inversion model results when using an initial model that is a halfspace of 10, 100, 300, and 1,000 Ωm, respectively. Panels (e) and (f) use layered initial models, based on averaging the 2-D model of Comeau et al. (2018a) and from averaging 1-D models below each site, respectively. The model in (b) is the preferred model; it fits the data best. Note these models use a vertical increase factor of 1.2, and a horizontal covariance parameter of 0.5. Boxes highlight main features. Details for each model are (starting model RMS misfit / number of iterations / final RMS misfit): (a) 12.25 / 96 / 2.186, (b) 17.22 / 120 / 1.588, (c) 27.75 / 96 / 1.936, (d) 51.98 / 86 / 2.008, (e) 33.16 / 94 / 2.031, (f) 19.54 / 88 / 1.824. Figure S2. Varying the horizontal covariance parameter for model L4000. Panels (a), (b), (c), (d), and (e) show the inversion model results for a horizontal covariance parameter of 0.3, 0.4, 0.5, 0.6, and 0.7, respectively. Large values smooth the model. Note the effect of the covariance is over the number of cells rather than the physical cell size, see Robertson et al. (2020). The model in (c) is the preferred model; it fits the data best. Note these models use a vertical increase factor of 1.2 (38 vertical cells), as well as a starting model of a 100 Ωm halfspace. The vertical covariance parameter was fixed, as in (a). Details for each model are as follows (starting model RMS misfit / number of iterations / final model RMS misfit): (a) 17.22 / 75 / 2.247, (b) 17.22 / 105 / 1.968, (c) 17.22 / 120 / 1.588, (d) 17.22 / 114 / 1.903, (e) 17.22 / 93 / 2.498. Figure S3. Varying the data included for model L4000. The inversion model results shown in panel (a) include full impedance (off-diagonal and diagonal components) and tipper data, (b) full impedance data (off-diagonal and diagonal components), and (c) the off-diagonal components (Zxy, Zyx) of t [file 40623_2021_1400_MOESM1_ESM.pdf]

Supporting Information for

Crustal architecture of a metallogenic belt and ophiolite belt: Implications for mineral genesis and emplacement from 3-D electrical resistivity models (Bayankhongor area, Mongolia)

Matthew J. Comeau[1], Michael Becken[1], Alexey V. Kuvshinov[2], Sodnomsambuu Demberel[3]

[1] Institut für Geophysik, Universität Münster, Corrensstrasse 24, 48149 Münster, Germany.
[2] Institute of Geophysics, Swiss Federal Institute of Technology (ETH), Sonneggstrasse 5, 8092 Zurich, Switzerland.
[3] Institute of Astronomy and Geophysics, Mongolian Academy of Sciences, P.O.B-152, 13343 Ulaanbaatar, Mongolia.

Contents of this file

Figures S1 to S5

Introduction

This supporting information represents a small selection of the various inversion model testing that was carried out. Here we vary two critical inversion parameters, the starting model, Figure S1, and the covariance parameter, Figure S2, in order to illustrate the possible variation of the inversion model and the model features. In addition, we show the changes that arise from inverting different combinations of the data components in Figure S3. Furthermore, we evaluate the differences between models with data fit curves and residuals in Figure S4 and Figure S5.

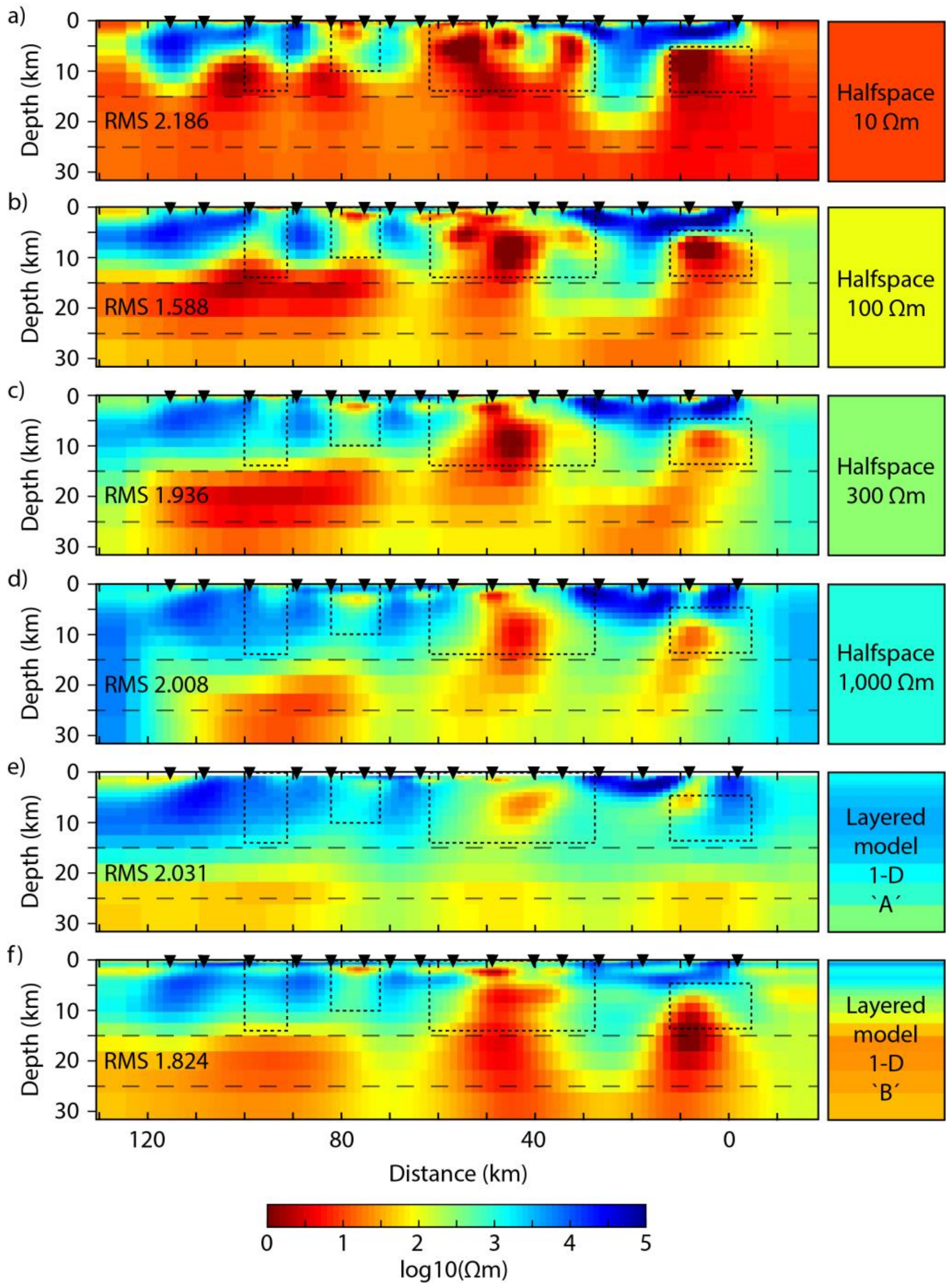

Figure S1. Varying the starting model for model L4000. Panels (a), (b), (c), and (d) show the inversion model results when using an initial model that is a halfspace of 10, 100, 300, and 1,000 Ωm, respectively. Panels (e) and (f) use layered initial models, based on averaging the 2-D model of Comeau et al. (2018) and from averaging 1-D models below each site, respectively. The model in (b) is the preferred model; it fits the data best. Note these models use a vertical increase factor of 1.2, and a horizontal covariance parameter of 0.5. Boxes highlight main features. Details for each model are (starting model RMS misfit / number of iterations / final RMS misfit): (a) 12.25 / 96 / 2.186, (b) 17.22 / 120 / 1.588, (c) 27.75 / 96 / 1.936, (d) 51.98 / 86 / 2.008, (e) 33.16 / 94 / 2.031, (f) 19.54 / 88 / 1.824.

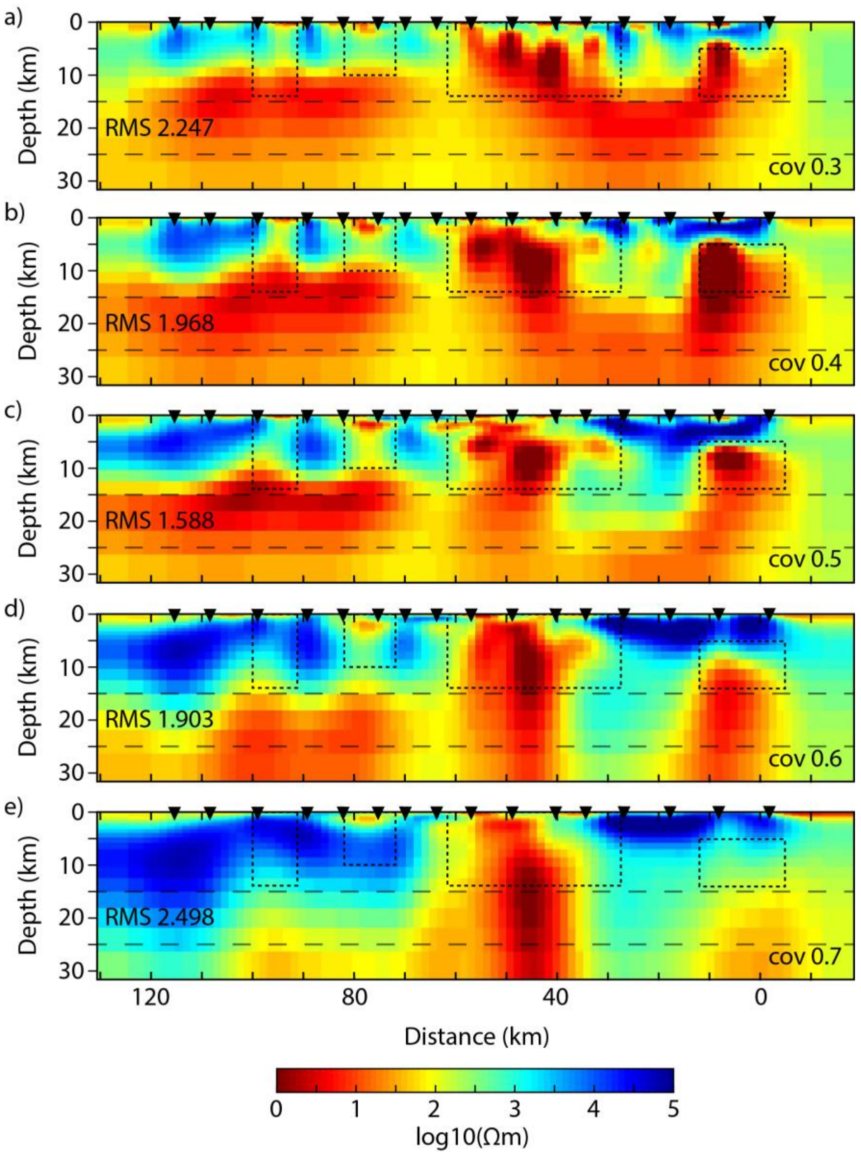

Figure S2. Varying the horizontal covariance parameter for model L4000. Panels (a), (b), (c), (d), and (e) show the inversion model results for a horizontal covariance parameter of 0.3, 0.4, 0.5, 0.6, and 0.7, respectively. Large values smooth the model. Note the effect of the covariance is over the number of cells rather than the physical cell size, see Robertson et al. (2020). The model in (c) is the preferred model; it fits the data best. Note these models use a vertical increase factor of 1.2 (38 vertical cells), as well as a starting model of a 100 Ωm halfspace. The vertical covariance parameter was fixed, as in (a). Details for each model are as follows (starting model RMS misfit / number of iterations / final model RMS misfit): (a) 17.22 / 75 / 2.247, (b) 17.22 / 105 / 1.968, (c) 17.22 / 120 / 1.588, (d) 17.22 / 114 / 1.903, (e) 17.22 / 93 / 2.498.

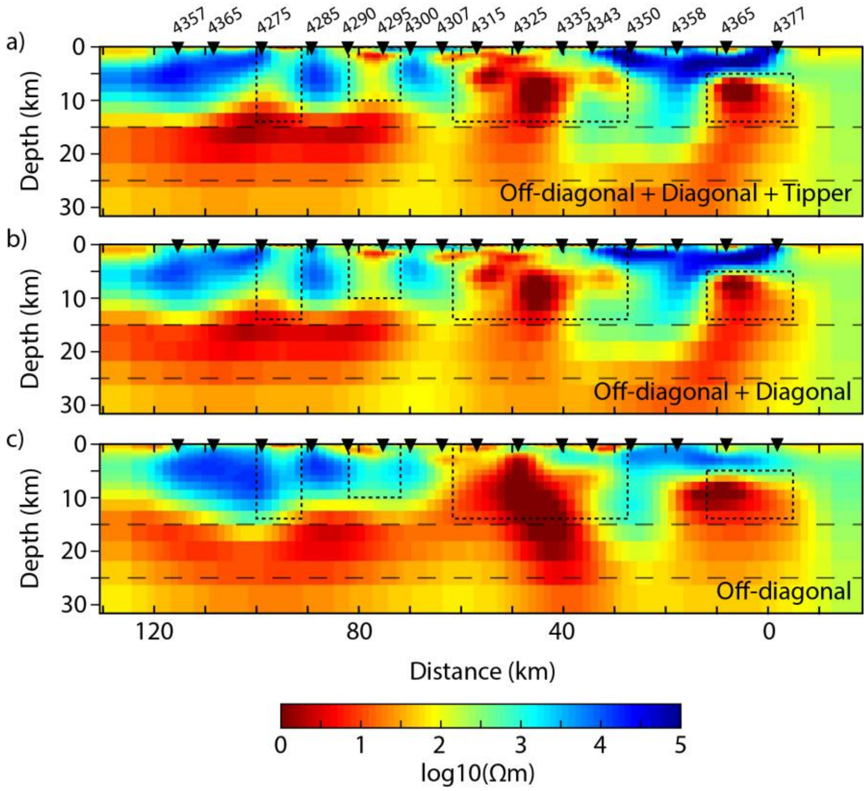

Figure S3. Varying the data included for model L4000. The inversion model results shown in panel (a) include full impedance (off-diagonal and diagonal components) and tipper data, (b) full impedance data (off-diagonal and diagonal components), and (c) the off-diagonal components (Zxy, Zyx) of the impedance only. Note these models use a vertical increase factor of 1.2 (38 vertical cells), a starting model of a 100 Ωm halfspace, as well as a horizontal covariance parameter of 0.5. It is clear that the inclusion of the diagonal components influences the model and modifies the shape and strength of some structures. However, the locations of the main model features (boxes), and their interpretations, do not change. Details for each model are as follows (starting model RMS misfit / number of iterations / final model RMS misfit): (a) 17.22 / 120 / 1.588, (b) 18.45 / 95 / 1.757, (c) 21.92 / 78 / 1.227.

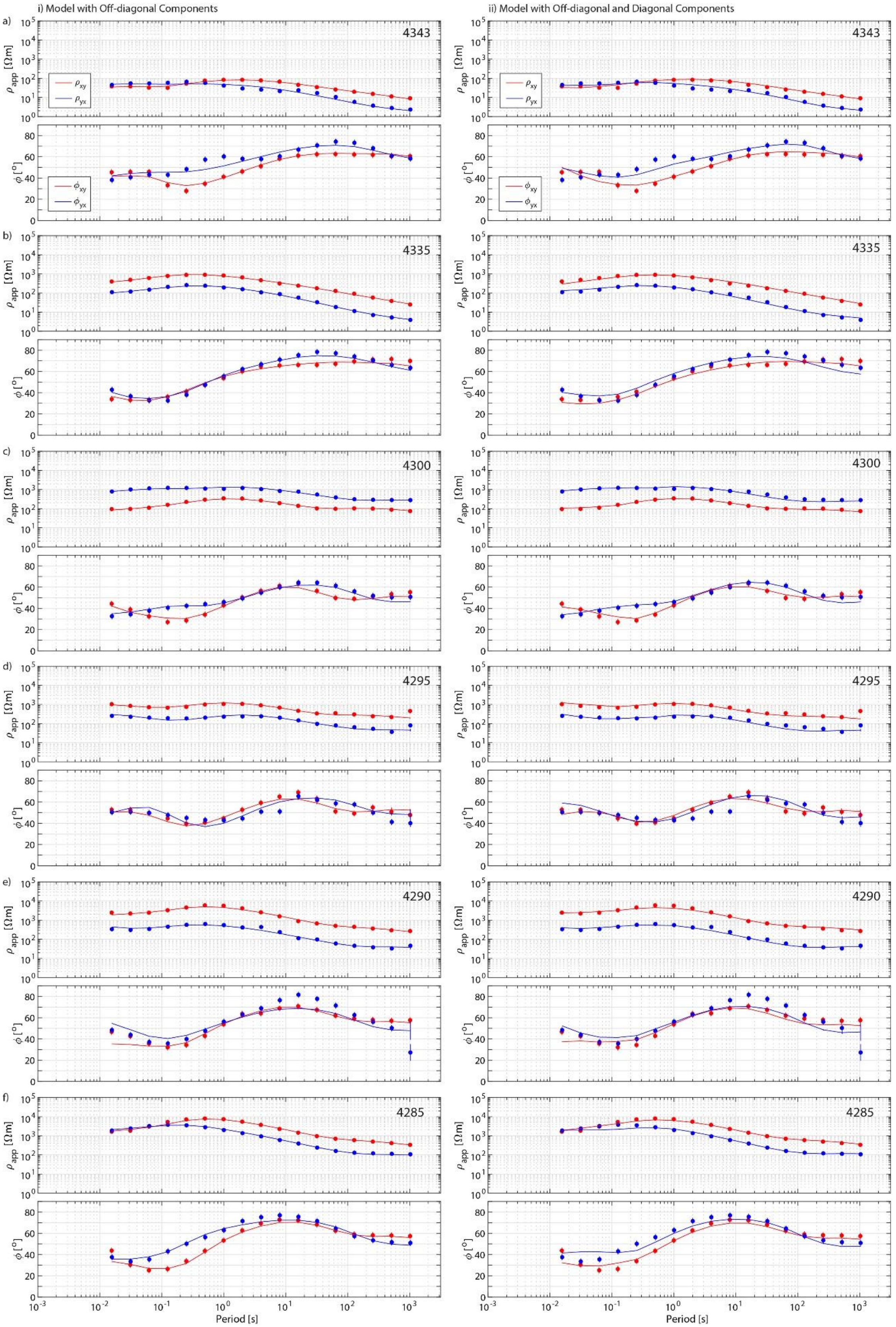
i) Model with Off-diagonal Components
ii) Model with Off-diagonal and Diagonal Components
a)
4343
b)
4335
c)
4300
d)
4295
e)
4290
f)
4285
ρ_{app} [Ωm]
ϕ [°]
ρ_{xy}
ρ_{yx}
ϕ_{xy}
ϕ_{yx}
Period [s]

Continued…

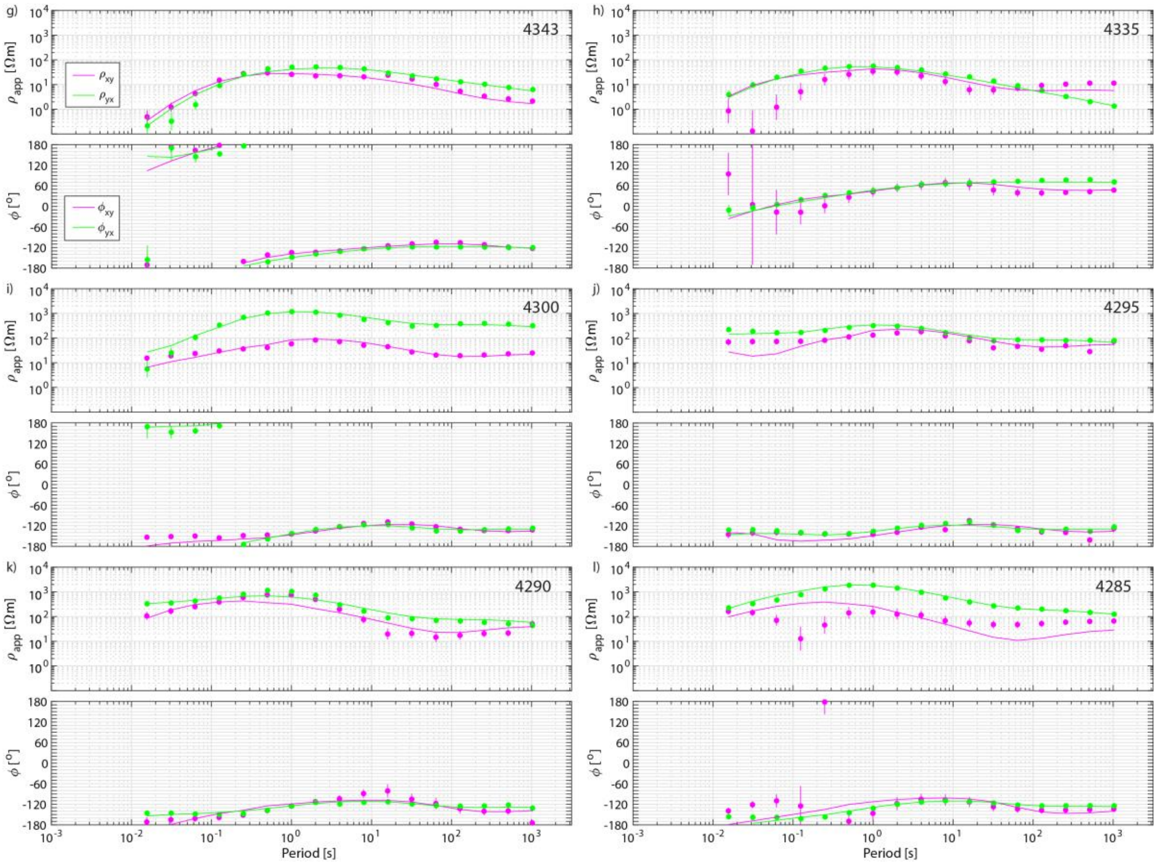

Figure S4. Comparing the fit of six sites for model L4000 (apparent resistivity, ρ_{app}, and phase, ϕ). Panels (a), (b), (c), (d), (e), and (f) correspond to sites 4343, 4335, 4300, 4295, 4290, and 4285 from the model (Figure S3c) that includes only off-diagonal components (column i) and the model (Figure S3b) that includes both off-diagonal and diagonal components (column ii). The corresponding diagonal components are shown in panels (g), (h), (i), (j), (k), and (l). The sites chosen are those above features of interest that appear to change between the two models (i.e., the fault/suture zone and the mineral belt). In both cases the fit of the model to the data is good. It is clear that the inclusion of the diagonal components influences the fit of the off-diagonal components.

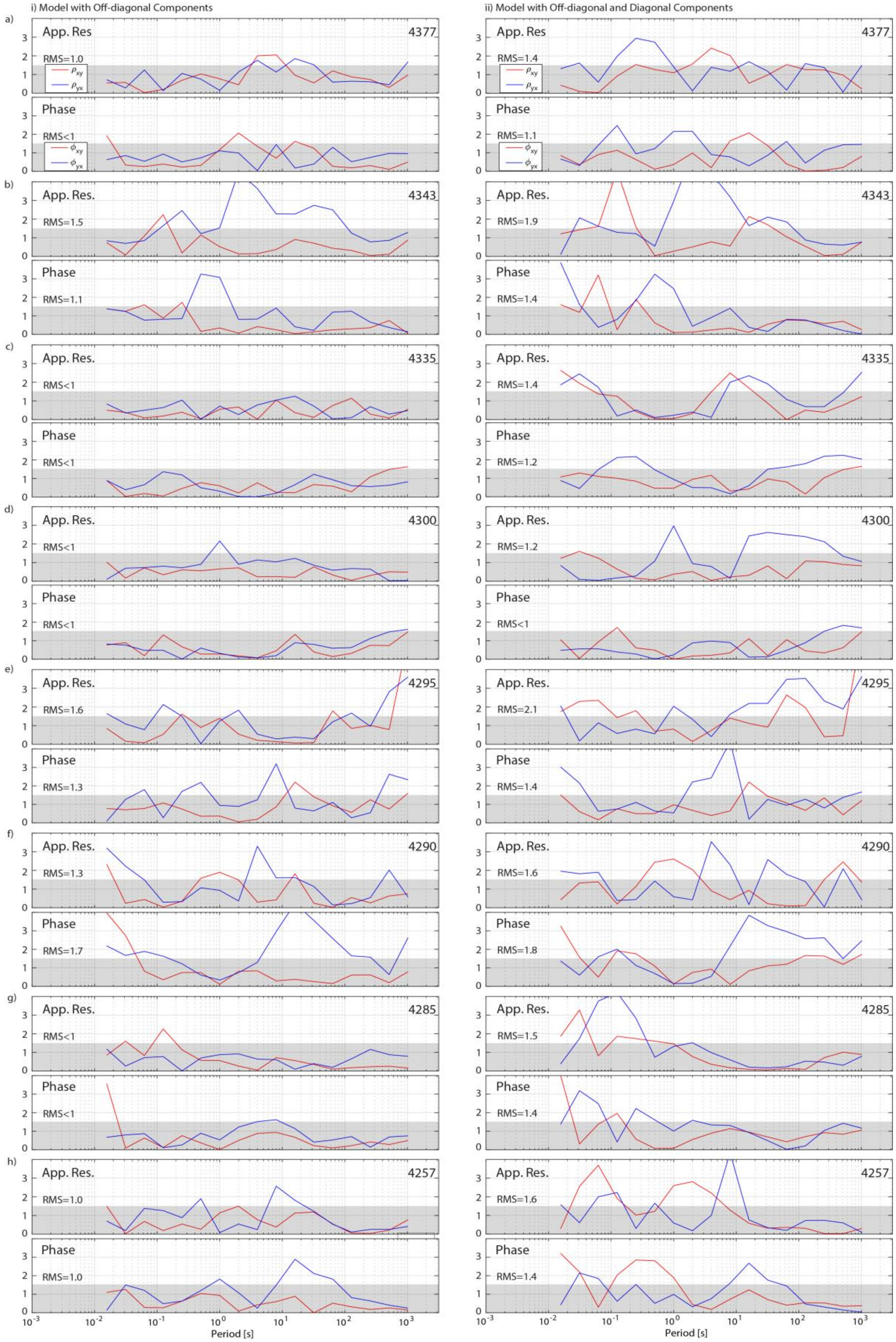
i) Model with Off-diagonal Components
ii) Model with Off-diagonal and Diagonal Components
a)
App. Res
4377
RMS=1.0
RMS=1.4
ρ_{xy}
ρ_{yx}
Phase
RMS<1
RMS=1.1
ϕ_{xy}
ϕ_{yx}
b)
App. Res.
4343
RMS=1.5
RMS=1.9
Phase
RMS=1.1
RMS=1.4
c)
App. Res.
4335
RMS<1
RMS=1.4
Phase
RMS<1
RMS=1.2
d)
App. Res.
4300
RMS<1
RMS=1.2
Phase
RMS<1
RMS<1
e)
App. Res.
4295
RMS=1.6
RMS=2.1
Phase
RMS=1.3
RMS=1.4
f)
App. Res.
4290
RMS=1.3
RMS=1.6
Phase
RMS=1.7
RMS=1.8
g)
App. Res.
4285
RMS<1
RMS=1.5
Phase
RMS<1
RMS=1.4
h)
App. Res.
4257
RMS=1.0
RMS=1.6
Phase
RMS=1.0
RMS=1.4
Period [s]

Figure S5. Error-normalized residuals of eight sites for model L4000. The residuals were computed as the absolute difference of the modelled data from the measured data for both apparent resistivity (top part) and phase (bottom part). Panels (a), (b), (c), (d), (e), (f), (g), and (h) correspond to sites 4377, 4343, 4335, 4300, 4295, 4290, 4285, and 4257 from the model (Figure S3c) that includes only off-diagonal components (column i) and the model (Figure S3b) that includes both off-diagonal and diagonal components (column ii). Note that sites 4377 and 4257 are at the ends of the model, not above the features of interest. The gray region indicates values <1.5, i.e. the absolute difference is less than 1.5x the error. The RMS misfit is given for each part. The residuals illustrate clearly that both models fit the data. Comparing the two models, it is obvious that there are differences in the residuals at all sites (not only those above the features of interest). Although the misfit is generally (slightly) higher at all sites for the model that includes both off-diagonal and diagonal components, the fit is still good.
